# Transcriptomic signals of mitochondrial dysfunction and OXPHOS dynamics in fast-growth chicken

**DOI:** 10.7717/peerj.13364

**Published:** 2022-05-04

**Authors:** Shawna Hubert, Giridhar Athrey

**Affiliations:** 1 Thoracic Head Neck Medical Oncology, MD Anderson Cancer Center, Houston, Texas, United States of America; 2 Department of Poultry Science, Texas A&M University, College Station, Texas, United States; 3 Faculty of Ecology and Evolutionary Biology, Texas A&M University, College Station, Texas, United States

**Keywords:** Fast-growth chicken, Oxidative phosphorylation, Aging, Oxidative stress, Birds, Longevity

## Abstract

**Introduction:**

Birds are equipped with unique evolutionary adaptations to counter oxidative stress. Studies suggest that lifespan is inversely correlated with oxidative damage in birds. Mitochondrial function and performance are critical for cellular homeostasis, but the age-related patterns of mitochondrial gene expression and oxidative phosphorylation (OXPHOS) in birds are not fully understood. The domestic chicken is an excellent model to understand aging in birds; modern chickens are selected for rapid growth and high fecundity and oxidative stress is a recurring feature in chicken. Comparing fast- and slow-growing chicken phenotypes provides us an opportunity to disentangle the nexus of oxidative homeostasis, growth rate, and age in birds.

**Methods and Results:**

We compared pectoralis muscle gene expression patterns between a fast and a slow-growing chicken breed at 11 and 42 days old. Using RNAseq analyses, we found that mitochondrial dysfunction and reduced oxidative phosphorylation are major features of fast-growth breast muscle, compared to the slow-growing heritage breed. We found transcriptomic evidence of reduced OXPHOS performance in young fast-growth broilers, which declined further by 42 days.

**Discussion:**

OXPHOS performance declines are a common feature of aging. Sirtuin signaling and NRF2 dependent oxidative stress responses support the progression of oxidative damage in fast-growth chicken. Our gene expression datasets showed that fast growth in early life places immense stress on oxidative performance, and rapid growth overwhelms the OXPHOS system. In summary, our study suggests constraints on oxidative capacity to sustain fast growth at high metabolic rates, such as those exhibited by modern broilers.

## Introduction

Birds are remarkable for their longevity despite their high metabolic rate. Birds show greater longevity than similarly sized terrestrial mammals (Costantini, 2008) – a life history trait associated with the maintenance of flying conditions until close to mortality (Ricklefs, 2010). At the cellular level, birds have unique evolutionary adaptations that may make them resistant to aging effects, for example, insensitivity to insulin and sensitization to glucagon that protects against DNA damage (Hickey et al., 2012). Oxidative stress, and accumulation of oxidative damage, are long-standing features of aging. Oxidative stress is the imbalance between the production of reactive oxygen species (ROS) and systemic antioxidant defenses, manifesting as free-radical mediated tissue damage (Betteridge, 2000).

Among birds, body mass correlates significantly with daily energy expenditure and resting metabolic rate, which is positively correlated with lifespan (Furness & Speakman, 2008). An organism’s metabolic rate is also linked with growth rate and feed efficiency - producing and converting energy to body mass. In the form of ATP, this energy is necessary for cell maintenance and replication and whole organism health and survival. The metabolic rate required for rapid growth rate is inversely related to feed efficiency. Higher metabolic rates elicit elevated heat production and oxygen consumption levels and decrease feed efficiency (Ojano-Dirain et al., 2007; Tallentire, Leinonen & Kyriazakis, 2018). Oxidative performance and mitochondrial function are central to organismal growth and fitness; ROS is also a regulator of mitochondrial biogenesis (Rasbach & Schnellmann, 2007) and energy homeostasis, which are together key to health and disease (Hinerfeld et al., 2004; Dai et al., 2010; Siegel et al., 2011).

**Figure 1:**
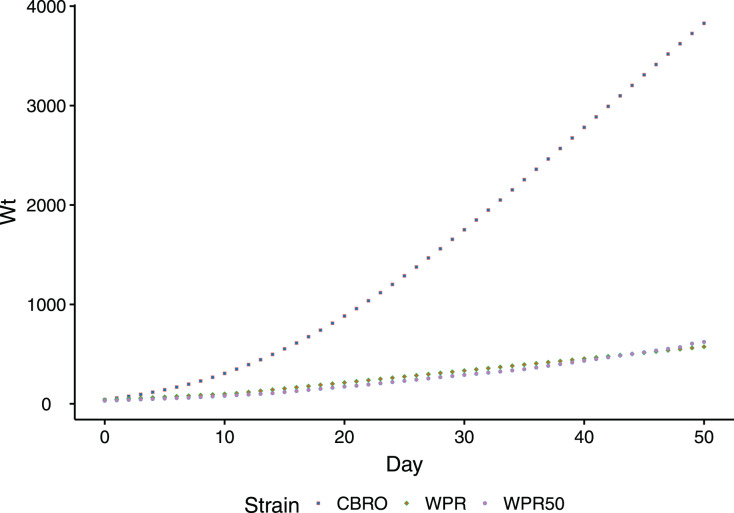
Comparison of body weight gain (growth rates) between CBRO and WPR. Growth curves depicting the change in body weight (y-axis, log scale) over the first 50 days (x-axis) of the life of a domestic chicken. The modern broiler (CBRO) grows to maximum weight over this period, whereas the unselected heritage strains will take over 2 years to reach the same weight. WPR and WPR50 are growth curves for the WPR strains that can be found today (WPR) or from the 1950s (WPR50).

The domestic chicken, *Gallus gallus*, is a unique model to study muscle oxidative performance in relation to growth rates, as divergent selection has generated strains with distinct growth/metabolic rate phenotypes. Modern broilers (meat-type chicken) are notable for their high metabolic rate (Scheele, 1997; Havenstein, Ferket & Qureshi, 2003), and their metabolism contributes directly to their fast growth performance. The growth rate of modern broilers rivals even the fastest-growing dinosaurs (Brunner et al., 2019). Compared to unselected chicken lines, which have changed little in a century, the growth rate of broilers is dramatically different (Fig. 1). Unselected domesticated chicken range in their growth phenotypes from smaller-bodied, slow growth birds (ancestral Red Junglefowl) to larger-bodied, slow growth birds (standard domestic strains).

Even though birds have endogenous defenses against oxidative damage, fast-growth chickens have a well-documented history of oxidative stress. Broilers experience oxidative stress arising from heat stress, negatively affecting feed efficiency, immune function, and welfare (Altan et al., 2003; Fellenberg & Speisky, 2006) (Gu, Hao & Wang, 2012; Huang et al., 2015). These phenotypes also have a high incidence of metabolic and skeletal disorders. Tickle, Hutchinson & Codd (2018) showed that the energy requirements of larger breast muscles place severe constraints on the broiler’s energy budget and compromise overall skeletal development and the efficiency of respiration. In the muscle disorder called wooden breast (WB), the main features are oxidative stress and mitochondrial dysfunction, leading to hypoxia, muscle fiber degradation, and infiltration of macrophages (Soglia et al., 2016; Abasht et al., 2016; Kuttappan, Hargis & Owens, 2016; Papah et al., 2017; Petracci et al., 2019). Several concomitant conditions, including WB, White Striping (WS), and Spaghetti Meat (SM), are unique to fast-growth, high-feed efficiency traits (Petracci et al., 2019).

### Oxidative phosphorylation

One long-standing evolutionary hypothesis relates mitochondrial dysfunction and oxidative stress to aging (Bandy & Davison, 1990). The mitochondria are responsible for most of the cell’s ATP production through oxidative phosphorylation (OXPHOS). The OXPHOS system comprises five protein complexes, with the subunits of each complex encoded by both mitochondrial and nuclear genes. Thirteen mitochondrial genes encode OXPHOS subunits, whereas the remaining (55–70) are encoded by nuclear genes (Smeitink, van den Heuvel & DiMauro, 2001; Reinecke, Smeitink & van der Westhuizen, 2009).

Defects in the OXPHOS system cause many pathologies (Shoffner et al., 1991; Zeviani et al., 1995; Leonard & Schapira, 2000; Chandra & Singh, 2011), and mitochondrial dysfunction and reduced OXPHOS performance can have multiple origins. The cause-effect relationships between mitochondrial dysfunction and aging remain unresolved (Kauppila, Kauppila & Larsson, 2017), but reduced OXPHOS performance and oxidative stress are common to various age-related disorders (Fannin et al., 1999; Peterson, Johannsen & Ravussin, 2012; Bratic & Larsson, 2013; Chistiakov et al., 2014; Mastroeni et al., 2017). Given the high prevalence of oxidative stress-related disorders in chicken, it is important to develop a deeper understanding of OXPHOS performance between fast and slow growth phenotypes.

While mitochondrial involvement has been of interest recently in muscle disorders of chicken (Papah et al., 2017), the mitochondrial dynamics and oxidative phosphorylation in relation to the growth rate is relatively less understood. OXPHOS defects in skeletal muscles underlie disorders of skeletal muscles (Wallace, 1992; Esposito et al., 1999; Kunz, 2001). Skeletal muscles are highly oxidative and energy-intensive, and OXPHOS is the most effective ATP supply mechanism. However, the pectoralis muscle in chicken is mainly type IIB (glycolytic) by 42 days (slaughter age), but the muscle transitions from type IIA (>99%, mitochondria rich, oxidative) to type IIB (glycolytic fast-twitch) around week four (Barnard, Lyles & Pizzey, 1982). Given this ontogenic transition, it is necessary also to consider OXPHOS performance in the context of these developmental stages.

The aim of this study was to investigate the oxidative performance in the pectoralis muscle of chicken in relation to the growth rate. Modern broilers have high resting metabolic rates, and even during inactivity, they have a higher metabolic rate than similarly sized galliform birds (Tickle, Hutchinson & Codd, 2018). As most of the metabolic demands of a broiler are driven by the economically important pectoralis muscle, we investigated gene expression differences between the pectoralis muscle between fast and slow growth broilers and illuminate the oxidative dynamics between growth phenotypes.

## Materials and Methods

### Birds and rearing conditions

We started the growth rate study with one-hundred and twenty hatch-day chicks (straight run) each of a fast-growth commercial broiler (Ross Strain, CBRO) and a slow-growth foundational broiler (White Plymouth Rock, WPR), 240 birds in total. We did not quantify resting metabolic rates in these strains, but there is an abundance of data on differences in feed conversion ratios in these breeds. We tracked growth performance by quantifying weekly body weight gain. We raised birds (experimental units) on identical, energy-equivalent diets. We provided feed and water *ad libitum*, and we performed welfare checks twice daily. We weighed the birds weekly and confirmed that the CBRO matched the growth profile expected by the primary breeding company. Unless birds had to be removed from the study due to illness, they were included in the analyses. We performed animal care and euthanasia procedures according to protocols approved by Texas A&M’s Institute for Animal Care and Use Committee (AUP IACUC 2016-0065).

### Tissue sampling and sequencing

To assess mitochondrial gene expression and OXPHOS dynamics in relation to age and growth performance (strain), we collected tissue samples from the anterior portion of the pectoralis major muscle at 11 and 42 days of age. We used the WPR as a baseline phenotype for assessing mitochondrial function. We used the foundational breed as the baseline instead of the ontogenic state to avoid confounding our results due to the transition in fiber type that occurs at 4 weeks of age. At each age, we euthanized 24 randomly-selected birds of each breed by CO_2_ exposure, followed by cervical dislocation, and tissues were collected and preserved. We dissected approximately two grams of breast tissue and stored it in RNALater (Ambion Inc., Austin, TX, USA) at a1:5 ratio. We stored breast tissue samples at 4 °C for a minimum of 24 h and then removed them from RNALater and stored them at −80 °C until RNA isolation. We collected 96 breast tissues for this study (2 breeds × 2 ages × 24 samples), and each sample was considered a biological replicate for the gene expression study.

We extracted total RNA from 100 mg sections of *Pectoralis major* tissue samples through the TRIzol Reagent method following the manufacturer’s protocol (Thermo Fisher Scientific, Waltham, MA, USA). We checked the concentration of RNA isolates on a Nanodrop spectrophotometer (Thermo Fisher Scientific, Waltham, MA, USA) and analyzed the quality and integrity using an Agilent Bioanalyzer 2100 (Agilent Technologies, Santa Clara, CA, USA) with the RNA 6000 Nano Kit. We kept only the samples with RNA Integrity scores (RIN) above eight for gene expression analysis using RNAseq. We also calculated the DNA content in our RNA isolates by fluorometric quantitation on a Qubit (Thermo Fisher Scientific, Waltham, MA, USA) and found that the DNA content was very low (<2%).

Next, we prepared mRNA libraries for transcriptome sequencing using the Lexogen QuantSeq 3′mRNA Library Prep Kit (Lexogen, Vienna, Austria). We generated 96 single-indexed libraries. We assessed the libraries for insert size distribution (100–200 bp) with the Agilent TapeStation D1000 DNA ScreenTape and estimated the library concentration using the Qubit dsDNA High Sensitivity Kit (ThermoFisher Scientific, Waltham, MA, USA). Of these, 72 libraries (*n* = 18/age/breed) were of sufficient quality and concentration and selected for sequencing. For sequencing, we pooled the single-indexed libraries in equimolar proportions and sequenced them in single-end mode (75 bp) on an Illumina NextSeq platform at the Texas A&M University Institute for Genome Sciences and Society (College Station, TX, USA). Based on a power analysis using the R package ssizeRNA (Bi & Liu, 2016), we determined that our design had over 99% power to detect differentially expressed genes at FDR = 0.05 and Log(Fold Change) of 2 (effect size).

### Differential expression and statistical analyses

To identify differential gene expression, we compared the breast transcriptomes between the slow-growth WPR and fast-growth CBRO varieties at 11- and 42-days. We analyzed the RNAseq data using a well-established pipeline based on the counting of uniquely mapped reads, followed by statistical analysis of normalized counts data. Briefly, we checked the raw Illumina sequence reads with FastQC (version 0.11.6) and MultiQC (version 1.4), followed by adapter trimming and quality filtering with Trim_Galore version 0.4.3 (Martin, 2011; Ewels et al., 2016; Babraham Institute, 2018a, 2018b). We discarded reads with average quality scores lower than Q30 and shorter than 30 bp. We aligned the quality-filtered data to the *Gallus gallus* reference genome (Version 4.8, Ensembl Release 85, downloaded July 2016) with the short-read de-novo splice mapper STAR (version 020201) (Dobin et al., 2013; Dobin & Gingeras, 2015) and counted the reads mapping uniquely to exons with HTseq-Count (version 0.9.1) (Anders et al., 2013; Herrero et al., 2016; Aken et al., 2016; Ruffier et al., 2017). We used the TMM normalized (trimmed mean of M-values) counts for each library to perform statistical analysis for differential expression using the EdgeR package in R (Robinson, McCarthy & Smyth, 2010; McCarthy, Chen & Smyth, 2012; Anders et al., 2013; RStudio Team, 2015; R Core Team, 2019). We verified the applicability of the negative-binomial distribution-based method by visualizing the Mean-Variance plot. We identified genes as significantly differentially expressed (FDR < 0.05) using the Quasi-likelihood F-test implemented in the ‘glmQLFtest’ function. We analyzed results for gene ontology and pathway analysis using the Ingenuity Pathway Analysis (IPA, Qiagen Inc, Germany) platform. To investigate mitochondrial dynamics, we selected the 37 genes encoded by the mitochondria and nuclear-MT counterparts that are part of the OXPHOS pathway; these include all the annotated OXPHOS pathway genes in chicken. We used the normalized LogCPM values for gene-by-gene comparisons of interest.

## Results

Two CBROs and one WPR were excluded from the study as a result of sudden death in the first week of the study. These mortalities did not affect the remainder of the study or our planned sampling.

**Figure 2:**
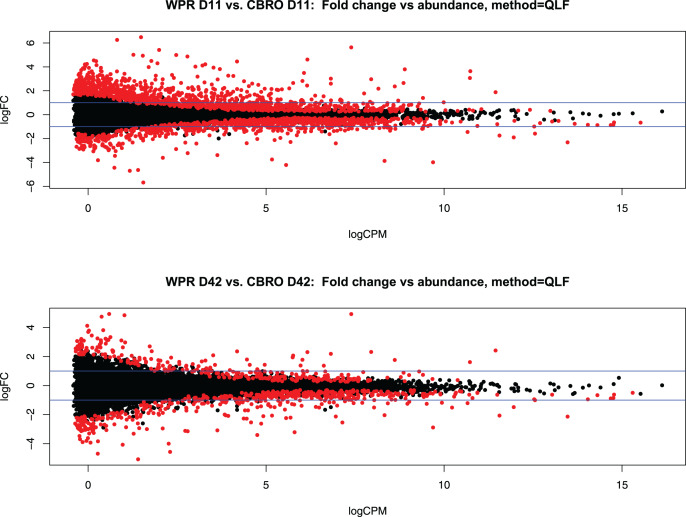
Representation of differential gene expression at D11 and D42. Mean-average (MA) plots showing the total differentially expressed gene (DEG) patterns between the D11 (A) and D42 (B) comparisons between WPR and CBRO strains. We observed 2,137 DEG at D11 and 667 DEG at D42. The colored points show the significantly differentially expressed genes. Values less than zero are downregulated in CBRO, and values greater than zero are upregulated in CBRO.

We sequenced 72 mRNA libraries in this study, generating 350 million reads, averaging 4.8 M quality-filtered reads per library. Differential gene expression analysis with EdgeR showed 2,136 differentially expressed genes (DEG) at 11 days (between WPR and CBRO) and 667 DEG at 42 days old, at the FDR < 0.05 level. In both comparisons, expressed transcripts mapped to coding genes, lncRNA, and pseudogenes, but all differentially expressed genes mapped to coding genes only. Figures 2A and 2B show the mean-average plots for these two comparisons. We also found a distinct clustering of expressed genes based on the growth rate and age classifications, with the CBRO samples, especially day 42 samples, forming a nearly uniform cluster in a single clade. Figure S1 shows a hierarchical clustering plot depicting the expression patterns of the top 50 most variable genes across samples.

For the WPR *vs* CBRO (D11), the enriched gene ontology terms (biological processes) were “Cellular Processes (GO:0009987)”, “Biological Regulation (GO:0065007)”, and “Metabolic Process (GO:0008152)” based on PantherDB, and the terms “Immune System Process” and “Immune Response” were significantly enriched based on gene list analysis. The top five differentially regulated genes (by FDR) in this comparison were Haemoglobin Subunit Epsilon 1 (HBE1), Glycine Amidinotransferase (GATM), Glutaredoxin 5 (GLRX5), Musculoskeletal Embryonic Nuclear Protein 1 (MUSTN1), and Pancreatic Progenitor Cell Differentiation and Proliferation Factor (PPDPF), of which MUSTN1 was the only upregulated gene.

For the day 42 comparison, the PantherDB based gene ontology terms were the same but in a different order (Cellular Processes, Metabolic Process, and Biological Regulation). Gene list enrichment showed the top functional categories to be “Drug Metabolic Process” and “Small Molecule Metabolic Process”. The top differentially expressed genes (by FDR) were GATM, Dynein Light Chain (BC048507), Methionine Adenosyltransferase 1A (MAT1A), Crystallin Alpha B (CRYAB), and Colony Stimulating Factor 2 Receptor Subunit Alpha (CSF2RA). The gene glycine amidinotransferase (GATM, ENSGALG00000023435) was one of the top differentially expressed genes at both D11 and D42. This gene encodes a mitochondrial gene involved in creatine biosynthesis. As creatine is an important energy transporter between the mitochondria and the cytosol, the implication of this gene in this context is highly relevant and notable. Deficiency of the GATM is associated with morbidities in humans, including myopathies.

### Canonical pathways

**Figure 3:**
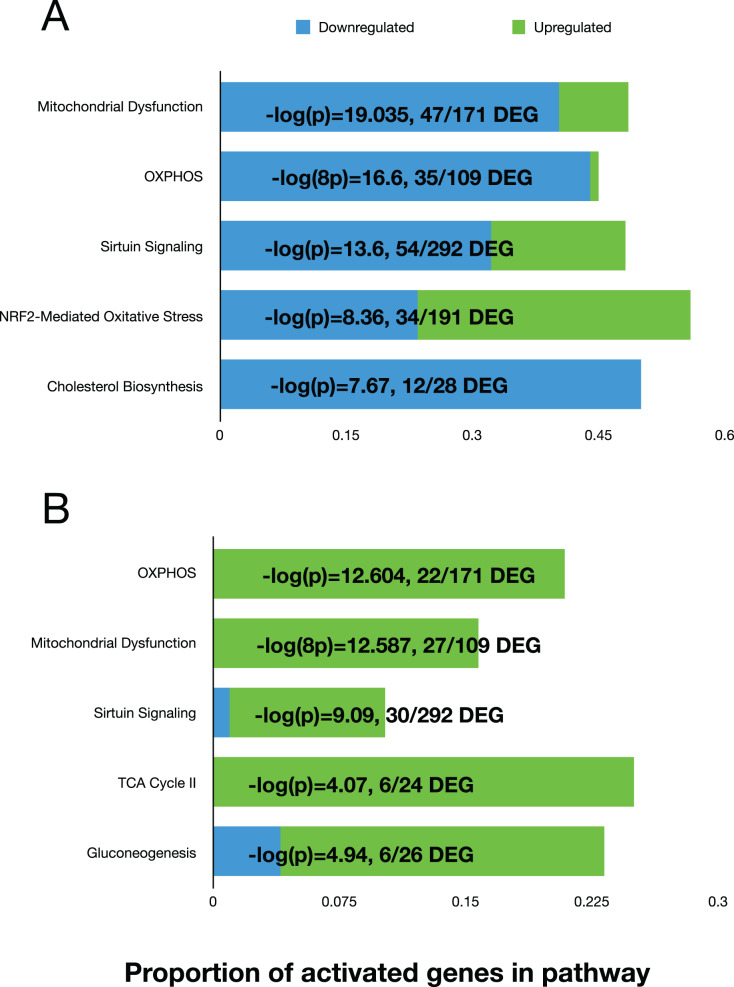
The top five activated pathways in the D11 and D42 comparisons. Bar plots showing the top five activated canonical pathways based on differential gene expression in the breast tissue between slow-growth WPR and fast-growth CBRO. (A) and (B) show these results for the D11 and D42 comparisons, respectively. The proportion of up and down-regulated genes within each pathway is shown and the −log(*P*) value that supported the ranking of these pathways.

We used the differential expression data to perform pathway analysis to determine activated canonical pathways and upstream regulators using the Ingenuity Pathway Analysis (IPA) software. The top five canonical pathways at 11 days, based on the Log(*P*) values, were ‘Mitochondrial Dysfunction’, ‘OXPHOS’, ‘Sirtuin Signaling’, NRF2-Mediated Oxidative Stress Response, and Cholesterol Biosynthesis (Fig. 3A). At day 42, the top five pathways were “OXPHOS”, “Mitochondrial Dysfunction”, “Sirtuin Signaling”, TCA Cycle II, and Gluconeogenesis (Fig. 3B). The ‘upstream regulator’ for the day 11 comparison was TP53 (Tumor Protein 53), but for the day 42 comparison, RICTOR (RPTOR independent companion of mTOR complex 2) was the top regulator. Both regulators are involved in various cell proliferative and regulatory functions. Overall, the top pathways showed that mitochondrial function was reduced, and sirtuin signaling was activated in fast-growth broilers. Due to the predominance of pathways and molecules involved in OXPHOS and mitochondrial function, we performed additional investigations into the OXPOS gene expression patterns.

### OXPHOS respiratory complexes

To further investigate the processes driving ‘Mitochondrial Dysfunction’ and ‘OXPHOS’ inhibition in CBRO, we investigated patterns of mitochondrial gene expression and the nuclear-mitochondrial encoded respiratory complexes. All 37 annotated mitochondrial genes were expressed in both strains at both ages. Of these, 13 mitochondrial genes encode the subunits of the respiratory complexes. We found that on day 11, all 13 mitochondrial subunit genes were differentially expressed between WPR and CBRO, whereas on day 42, 11 of the 13 were differentially expressed.

**Figure 4:**
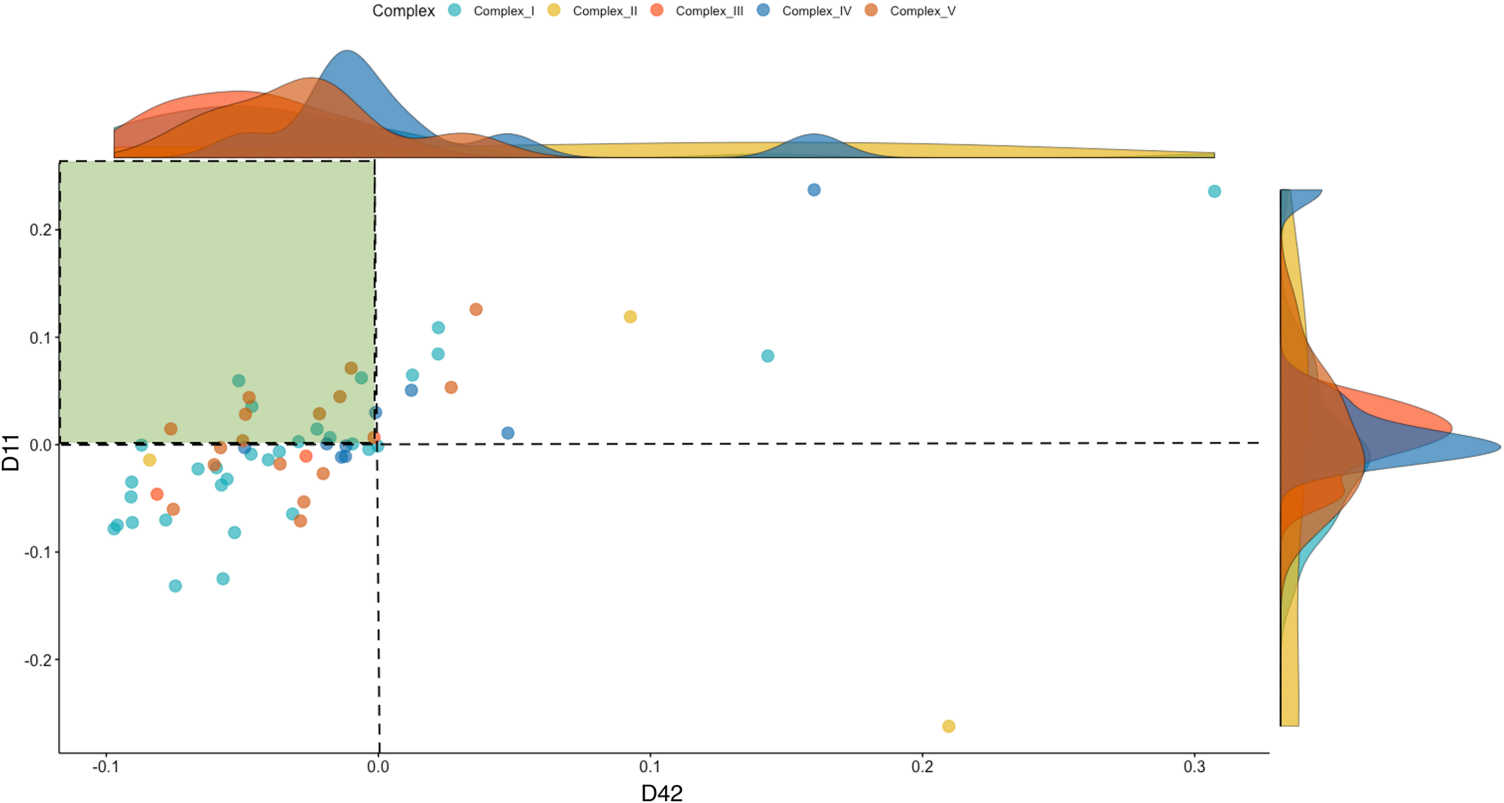
Expression of OXPHOS genes in the CBRO standardized against the age-matched WPR group. A plot showing the change in expression of the mitonuclear genes of the OXPHOS respiratory complexes between Day 11 (y-axis) and Day 42 (x-axis). Each point represents the expression intensities (Log CPM) in the CBRO strain, represented as a percentage of the LogCPM from age-matched WPR strain. Values less than zero show genes that have lower expression in CBRO relative to WPR. The genes in the green-shaded area showed a significant reduction in expression intensities at Day 42, compared to Day 11. Overall, OXPHOS performance declined 37% between D11 and D42. The density plots on the boundary of the plot show the density of expression intensities by complex. Complexes III, IV and V show the most notable downward shifts in expression at D42, based on the shape and position of the density plots.

To investigate the dynamics of the OXPHOS system, we separated the mitonuclear coding genes that comprise the five respiratory complexes (Complex I–V) from the total genes. These included 52 named OXPHOS orthologs from the chicken nuclear genome (ENSEMBL Release 85), besides the 13 mitochondrial coding genes. To characterize the directionality of the differences in OXPHOS between WPR and CBRO, we represented the normalized expression intensities (log counts per million, LCPM) of the CBRO mitochondrial coding subunits as a proportion of the WPR expression intensities. Overall, the median expression of OXPHOS genes in CBRO on day 11 was 13% lower than the slow-growth WPR strain but declined further (21% lower) by day 42. The distribution of expression values between D11 and D42 CBRO was significantly different (Kolmogorov-Smirnov test D = 0.317, *P* = 0.001). Using the slow-growth OXPHOS expression intensities (age-matched) values as the baseline level, we found that OXPHOS expression in CBRO declined between D11 and D42 (Fig. 4, Wilcoxon rank sum test, W = 2,593, *P* = 0.0032). The median differences showed an effect size of 0.376 (Cohen’s D), a medium-sized effect. The range of expression intensities of these genes across the four groups (WPR D11, WPR D42, CBRO D11 and CBRO D42) are shown in a Cleveland Dot Plot in Fig. S2, and shows the same pattern. In summary, we found that OXPHOS performance, based on transcript activity, was diminished in CBRO relative to WPR, it also declined significantly between D11 and D42.

### Sirtuin signaling

The sirtuin signaling pathway was the next significant canonical pathway, with 80 out of 292 genes activated in the pathway. Notably, none of the sirtuin family genes were differentially expressed. Besides genes that overlap with the OXPHOS pathway, other notable downregulated genes were superoxide dismutase 1 (SOD1) and SOD2. Superoxide dismutase genes encode enzymes involved in ROS detoxification.

### Comparison of D11 and D42 patterns

We used the comparative analysis approach in IPA to identify common themes between the D11 and D42 analyses. We found that Oxidative Phosphorylation was the top activated canonical pathway (Z-score = −5.916, P_BH_ = 5.59 × 10^−15^)_._ The top five common molecules (HBE1, GATM, GLRX5, MUST1, and PPDPF) also showed an interesting pattern in that they were significantly downregulated at D11, but were significantly upregulated at D42.

## Discussion

In this study, we compared empirical data from two distinct phenotypes, and relate growth rate to oxidative stress. The negative consequences of oxidative stress and damage in poultry are well documented (Fellenberg & Speisky, 2006; Surai et al., 2019). Oxidative stress in chicken arises from various technological, nutritional, environmental, or endogenous reasons (Soleimani et al., 2011; Salami et al., 2015; Rehman et al., 2018; Surai et al., 2019). In this study, we investigated the oxidative performance across ages in relation to early life growth rates using RNAseq. The activation of mitochondrial dysfunction, oxidative phosphorylation, and sirtuin signaling validate the molecular signatures of oxidative stress in the fast-growth phenotype, which is well documented in broilers. Our study is unique in assessing the dynamics of oxidative stress over time in chicken and provides two valuable insights into the relationship between growth rate, oxidative stress, and, potentially, longevity.

First, as we analyzed two divergent growth phenotypes of the same species, our dataset represents a ‘natural experiment’ that controls for genetic background and filters out differences arising from genetic stratification in cross-species comparisons. Hence, our results provide an understanding of how directional selection on growth rate may affect life-history traits such as longevity, which are linked to antioxidant mechanisms. The cellular processes and consequences accompanying aging are conserved across diverse organisms (Partridge & Gems, 2002; Kenyon, 2010; Flatt & Partridge, 2018). The length of the developmental period is inversely correlated with maximum lifespan, with slower growth reducing oxidative stress (Gavrilov & Gavrilova, 2001; Metcalfe & Monaghan, 2003). Fast growth is correlated with increased oxidative stress and reduced investment in homeostatic maintenance and repair (Metcalfe & Monaghan, 2003). Modern broilers today reach maximum size by 50 days, as opposed to the foundational breeds, which require over 2 years to reach the same body weight. Rapid growth before reaching sexual maturity reduces the antioxidant capacity in various bird species; conversely, slower-growing birds can allocate greater resources to somatic maintenance and repair (Gavrilov & Gavrilova, 2001; Vágási et al., 2018). The basal metabolic rate of high feed-efficiency broilers was 19% higher than in unselected lines in 1977 (Pym & Farrell, 1977), and modern broilers today add body mass faster than some dinosaurs (Brunner et al., 2019).

A second inference from our study is the implication for rapid growth preceding sexual maturity. Although we did not consider sexual maturity as an ontogenic checkpoint for sampling, the timeline for reaching the final adult body mass is one of the most dramatic differences between slow- and fast-growth broilers. In chickens, both age and body weight control sexual maturity, both in broilers and layers (Dunnington & Siegel, 1984), typically around 6 months from hatch. However, broilers reach maximum body weight by 8–9 weeks of age, well before sexual maturity (average 24 weeks). Unselected chicken lines eventually reach the same body size as modern broilers, but after sexual maturity, showing that growth rate, and not final body size, is the key trait that has consequences for oxidative stress. Buttemer, Abele & Costantini (2010) posited that similar-sized animals with different maximum lifespans differ in the amount of ROS per unit of oxygen consumed. While we did not measure ROS production in this study, our transcriptomic data suggest mitochondrial dysfunction and declining OXPHOS capacity over a short period. Both are characteristic of ROS-induced damage (Cui, Kong & Zhang, 2012; Chistiakov et al., 2014; Srivastava, 2017).

The slow pre- and postnatal developmental program in birds is important for maintaining ‘youthful’ conditions until close to mortality (Ricklefs, 2010), and retaining flight performance until mortality calls for the maintenance of physiological health until late in life. Slower development also delays immune activity, with increases inflammation with age. Fast-growth broilers have an accelerated postnatal developmental program, which is accompanied by a peak immune performance by the fourth week, in contrast to slower-growing birds (Cheema, Qureshi & Havenstein, 2003a, 2003b). The heightened immune activity in young broilers is necessary for the extrinsic threats of infection these birds may face in their domesticated environments, but the invocation of immune activity and inflammation early may also alter the energy metabolism of broilers. This supposition needs to be further investigated.

Our data also showed the activation of the sirtuin signaling pathway. Sirtuins are a family of highly conserved NAD+ enzymes important in the post-translational regulation of various metabolic processes (Houtkooper, Pirinen & Auwerx, 2012; Ren et al., 2017). Sirtuins regulate age-based gene expression - especially those associated with sexual maturity, aging, and senescence (Verdin et al., 2010; Lee et al., 2019; Surai et al., 2019). Fast growth broilers experience a high frequency of pathologies ranging from increased ascites, congestive heart failure (Nain et al., 2008), leg weakness (Paxton et al., 2013) and osteoporosis (Scheele, 1997; Julian, 2005). The activation of sirtuin signaling in this study against the backdrop of mitochondrial dysfunction raises the possibility that increased oxidative stress may affect the so-called longevity-assurance processes (Rattan, 1998), manifesting as aging-related pathologies.

While oxidative stress can be detrimental, avian species have evolved several endogenous antioxidant mechanisms (thioredoxin, glutathione, and coenzyme-Q). However, these antioxidant mechanisms are only efficacious against moderate stress (Surai et al., 2019). The transcriptome-based pathways suggest oxidative stress beyond the capacity of remedy *via* endogenous mechanisms. The NRF2 mediated oxidative stress response pathway, which was one of the top five activated pathways, is a mediator of antioxidant activity induction (Vomund et al., 2017; Surai et al., 2019). Dysregulation of the NRF2 pathway is a key target of various anti-inflammatory and anti-oxidative stress approaches. Our data suggest there may be limits to the effective antioxidant responses that can be sustained with a high growth rate (and mass-specific metabolic rates). Additional empirical data are required to determine exactly how antioxidant activities are affected. Whether this can be generalized to all birds beyond fast growth chicken needs further investigation.

Our data across two developmental windows show that rapid breast muscle growth in CBRO elicits a clear transcriptomic signature of oxidative stress in fast-growth broilers, with declining oxidative performance over short intervals. This result resembles findings in rats that experienced oxidative stress and reduced lifespan during rapid postnatal growth (Tarry-Adkins et al., 2016), but in that instance, maternal malnutrition was a contributing factor. While broiler breeders are not malnourished, they are feed-restricted to maximize reproductive performance. We did not investigate maternal factors in this study, but their contribution to the oxidative stress phenotype in broilers provides a potential avenue for managing oxidative stress in broilers.

The heat shock response (HSR) is among the common contributors to oxidative stress. However, our data did not show the induction of multiple heat-shock protein (HSP) genes. This result shows that thermal stress was not a causative factor in the oxidative stress observed in the experimental birds. This is an important result in our study, as heat stress is a well-known contributor to oxidative stress in chicken (Huang et al., 2015). However, we found that two of the cytosolic SOD genes were significantly downregulated - a pattern observed under high stress, where SOD is downregulated following apoptosis. The modulation of SOD activity is a tool for disease management (Surai, 2016), and from a translational standpoint, it would be valuable to investigate SOD dynamics across ontogenic stages in birds.

One of the key takeaways from our study is the decline in OXPHOS transcriptional activity over the lifespan of a fast-growth broiler, which highlights the genes and molecules invoked during oxidative stress in birds. The differential expression seen among mitonuclear complexes over a relatively short developmental period shows that muscle physiological processes dependent on oxidative performance are significantly altered between D11 and D42. The decline in OXPHOS activity, taken together with the activation of sirtuin signaling, is associated with senescence. Future studies need to determine if and how growth rates relate to senescence. Furthermore, additional data is required to characterize whether transcriptomic signals of senescence are associated with accumulation of damage at DNA, protein, and repair systems, as occurs in aging. To our knowledge, this is the first report of diminished OXPHOS capacity in young (D11) broilers and the first report providing empirical transcriptomic evidence for a decline in OXPHOS performance over time in fast growth broilers.

## Conclusions

In this article, we described evidence for oxidative stress dynamics in fast-growth broilers compared to slow growth chicken. We found that OXPHOS performance was significantly reduced in fast-growth broilers compared to the control, slow-growth chicken. We found the activation of specific aging-related pathways, all of which suggest transcriptomic signals of accelerated aging in fast-growth broilers. The early onset of molecular signatures of aging very early in the fast growth phenotypes shows not only the role of fast growth in generating oxidative stress but also highlighted the importance of oxidative stress to the health and welfare of chicken. Overall, our data provide insights into avian oxidative stress response systems under the demands of rapid growth during early life. This relation between rapid growth in early life and oxidative stress suggest evolutionary constraints regulating longevity in birds.

## Supplemental Information

10.7717/peerj.13364/supp-1 Supplemental Information 1 Similarity and clustering of samples among experimental groups. A heatmap showing the top fifty most variable genes across the replicates. A hierarchical clustering dendrogram shows the similarity of sampling within our experimental groups, and notably the tight clustering of CBRO D42 samples.

10.7717/peerj.13364/supp-2 Supplemental Information 2 Range of expression intensities of mitochondrial genes among experimental groups. A Cleveland Dot Plot showing the range of expression intensities for each of the mitochondrial genes (x-axis) that comprise the OXPHOS subunits. The Y-axis of the plot represents the expression values in terms of average LogCPMs. While expression was comparable across the experimental groups, several genes significantly differentially expressed, and show greater LogCPM values differing by 2 or more.

10.7717/peerj.13364/supp-3 Supplemental Information 3 Author Checklist.

## Additional Information and Declarations

### Data Availability

The following information was supplied regarding data availability: The sequence data are available at NCBI SRA Bioproject: PRJNA816173.
