# Supplementary figures and images for: Transcriptomic signals of mitochondrial dysfunction and OXPHOS dynamics in fast-growth chicken
Source: PeerJ. 2022 May 4;10:e13364. doi: 10.7717/peerj.13364 (PMC9078135; doi:10.7717/peerj.13364)

Color Key  
and Histogram

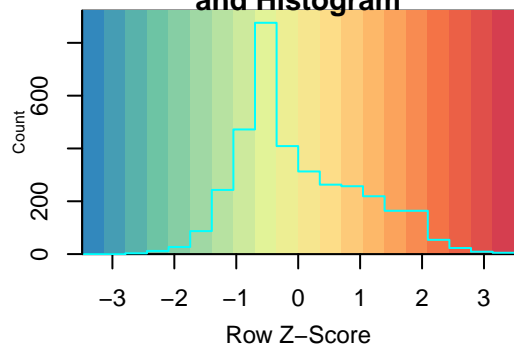

Top 50 most variable genes across samples

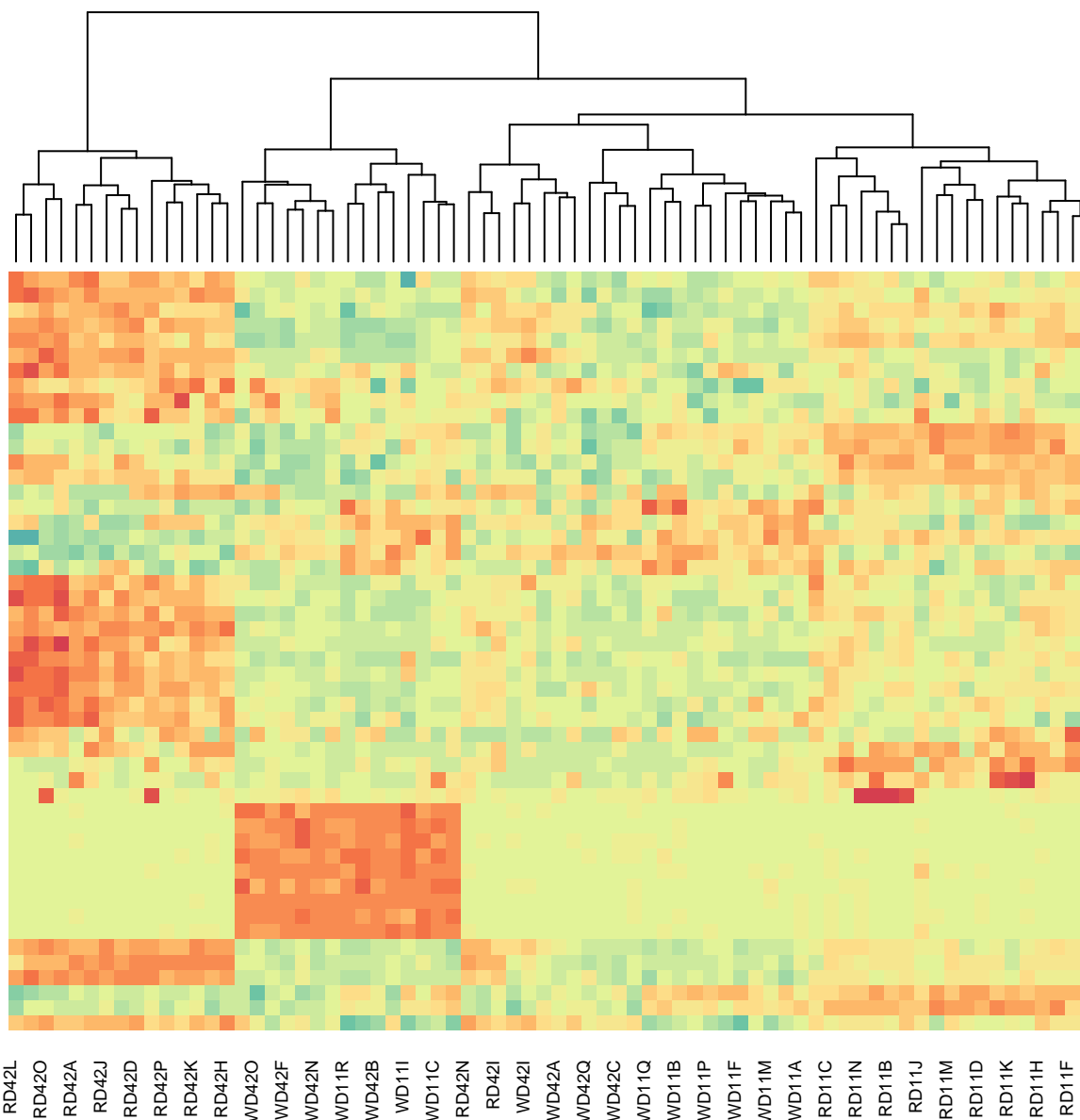

Supplement: Supplemental Information 1 — A heatmap showing the top fifty most variable genes across the replicates. A hierarchical clustering dendrogram shows the similarity of sampling within our experimental groups, and notably the tight clustering of CBRO D42 samples. [file peerj-10-13364-s001.pdf]

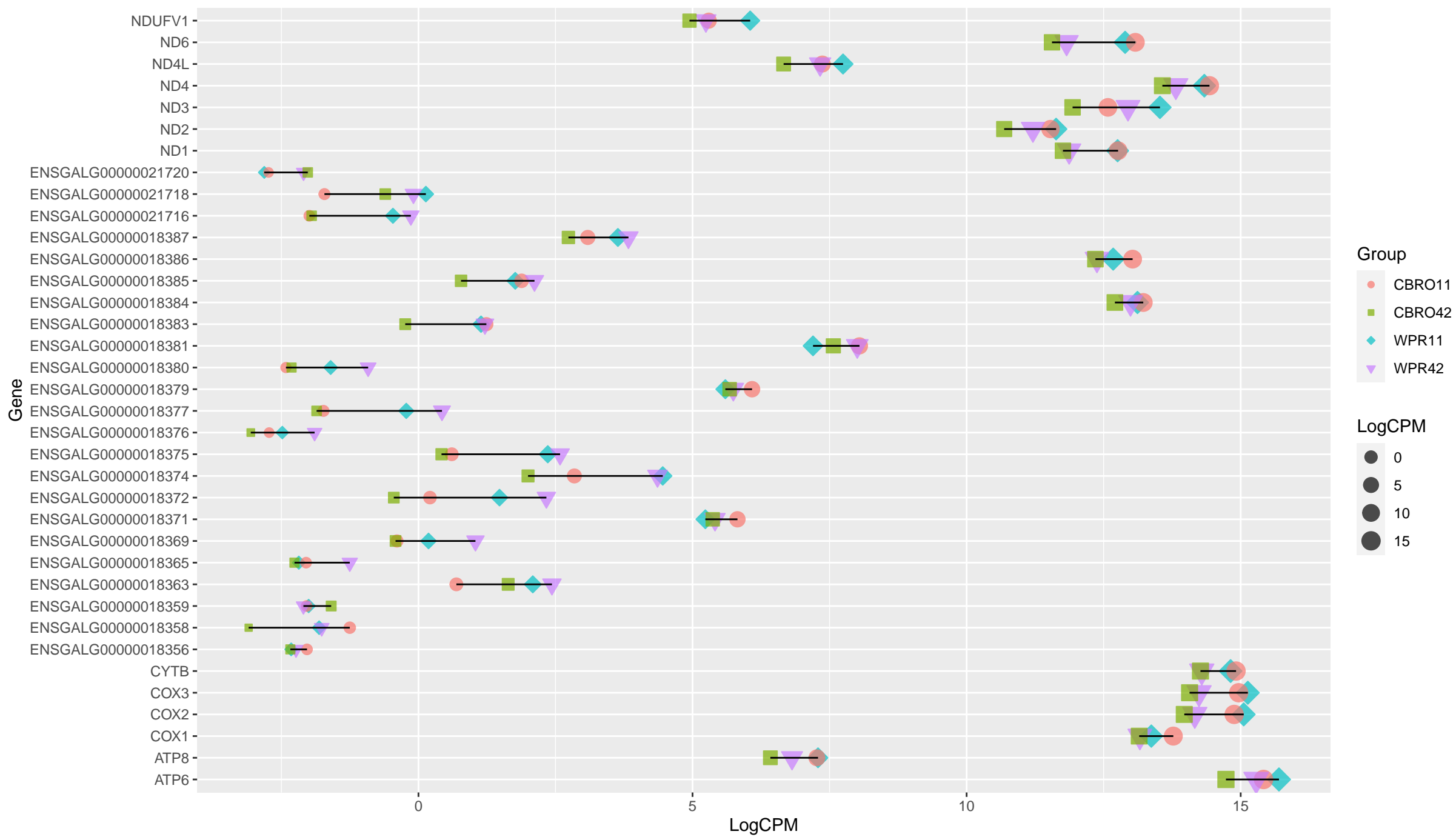

Supplement: Supplemental Information 2 — A Cleveland Dot Plot showing the range of expression intensities for each of the mitochondrial genes (x-axis) that comprise the OXPHOS subunits. The Y-axis of the plot represents the expression values in terms of average LogCPMs. While expression was comparable across the experimental groups, several genes significantly differentially expressed, and show greater LogCPM values differing by 2 or more. [file peerj-10-13364-s002.pdf]
